# Long-Term Neurodevelopmental Outcomes After Preterm Birth

**DOI:** 10.5812/ircmj.17965

**Published:** 2014-06-05

**Authors:** Farin Soleimani, Farzaneh Zaheri, Fatemeh Abdi

**Affiliations:** 1 Pediatric Neurorehabilitation Research Center, University of Social Welfare and Rehabilitation Sciences, Tehran, IR Iran; 2 Department of Midwifery, Kurdistan University of Medical Sciences, Sanandaj, IR Iran; 3 Students Research Committee, Shahid Beheshti University of Medical Sciences, Tehran, IR Iran

**Keywords:** Neurodevelopment, Impairment, Preterm Birth, Low Birth Weight

## Abstract

**Context:**

All over the the world, preterm birth is a major cause of death and important neurodevelopmental disorders. Approximately 9.6% (12.9 million) births worldwide are preterm.

**Evidence Acquisition:**

In this review, databases such as PubMed, EMBASE, ISI, Scopus, Google Scholar and Iranian databases including Iranmedex, and SID were researched to review relevant literature. A comprehensive search was performed using combinations of various keywords.

**Results:**

Cerebral palsy especially spastic diplegia, intellectual disability, visual (retinopathy of prematurity) and hearing impairments are the main neurodevelopmental disorders associated with prematurity.

**Conclusions:**

The increased survival of preterm infants was not associated with lower complications. There is now increasing evidence of sustained adverse outcomes into school age and adolescence, for preterm infants.

## 1. Context

Preterm birth is a major cause of death around the world and is one of the major health problems. Approximately 9.6% (12.9 million) births worldwide are preterm. The prevalence of preterm and low birth weight (LBW) births in United States is estimated at 8.2% per year (1) which includes a large proportion of deaths and causes short and long-term complications such as neurodevelopmental disorders (2, 3). Nearly 19000 infant deaths each year in Iran occurs due to premature birth, LBW, and other complications (4).

Overall, from 121 million neonates born each year, almost 23 million have LBW and the majority of them are in developing countries and cause by premature birth (5). LBW prevalence in Iran, according to the latest statistics have been reported as 9.6% to 11.8% (6, 7). The incidence of LBW also varies in other countries. It had a decreasing course between 1990 to 2010 in Estonia and Croatia, 2000 to 2010 in Sweden and The Netherlands, and 2005 to 2010 in Lithuania and Estonia. In the United States, the incidence showed an accelerated declining trend during the years 1989 to 2004 (8, 9). It is clearly shown that underweight infants suffer two to three times more than other infants from disabilities, health problems, and short and long-term psychological and social problems (10, 11).

In recent years, with advances in prenatal care and starting neonatal intensive care units, the survival rate of premature infants, low and very low birth weight (VLBW) has increased significantly (12, 13). However, the increased survival rate of premature infants was not associated with lower complications, and those infants who survived suffered more intensely from severe disabilities, intellectual disability, cerebral palsy, and hearing and visual impairments (14). Other studies suggest that there is little evidence regarding the association of increased disabilities with increased survival, and the overall rate of moderate to severe disability had been significantly decreased (15, 16). In Iran, the survival rate of LBW and VLBW infants were also reported as 98.4% and 66.6%, respectively (6).

Premature infants are at risk of major and minor deficits, such as cerebral palsy, cognitive and speech delays, motor and visual deficits, psycho-social and behavioral disorders, and dysfunction at school (17-21). This article aimed to review the developmental outcomes of preterm birth.

## 2. Evidence Acquisition

Data of this review were collected from our previous studies plus various data such as PubMed, EMBASE, ISI Web of science, Scopus, Google Scholar and Iranian databases including Iranmedex, and SID. A comprehensive search was performed using the combinations of the keywords “neurodevelopment, impairment, preterm birth, low birth weight” to review the relevant literature. The searches were performed using Boolean operators OR, AND between main phrase and the mentioned keywords were extracted from specific themes of the topic under study. A search strategy was built applying advanced search capability of the search engines. Based on this search strategy, only those articles were retrieved that had one of the first four keywords either in the title or the abstract. This strategy retrieved 150 articles. The inclusion criteria was set out in a way that only articles that explicitly dealt with neurodevelopmental outcomes after preterm birth were included. We also looked at the reference list of the retrieved papers and searched other search engines. A total of 150 articles were found in the primary search but after elimination of duplicates or irrelevant papers, only 79 records remained to be reviewed. The rational for selecting these articles were their popularity among researchers in the field. All published data from 1999 to 2013 is included in this review.

## 3. Results

### 3.1. Major Neurodevelopmental Disorders

The main disorders associated with prematurity include cerebral palsy especially spastic diplegia, intellectual disability, hearing loss, and visual impairment associated with retinopathy of prematurity. These impairments can occur together or separately during developmental periods, and they are sometimes complicated by progressive hydrocephalus or chronic seizures (22). They are usually symptomatic in the first two years of life; and their degree of severity may vary from mild to severe. A comprehensive and systematic approach for assessing the severity and impact of the disorder on daily function of children is required; implementing programs for early intervention and providing special education plans for these children is important (23).

These disabilities are 2 to 3 times more likely to occur in LBW infants compared to normal weight infants, and its prevalence increases with a more prametuarity and lower weight at birth birth (11, 24, 25). The rate of disabilities is higher in boys (16). A research showed in a study on developmental outcomes of VLBW infants with a report of neurosensory and neuro-functional status of them that compared to other infants, they have more neurological problems and developmental delays (26). Researchers of another study on 7500 infants in Iran concluded that the most common risk factors in developmental disorders for infants were prematurity, LBW, neonatal seizures, hyaline membrane disease, infection during pregnancy, and severe neonatal jaundice. In addition, it was found that developmental delay was also associated with factors such as parental consanguinity and abnormal fetal head circumference (26). Another study determined the factors significantly correlated with impaired motor development as perinatal asphyxia (with evidence of neonatal seizures, fetal distress and Apgar score of 0-3 at 20 minutes), LBW, preterm birth, and premature rupture of membranes (27).

#### 3.1.1. Cerebral Palsy

Cerebral palsy with various intensities and types is the most common developmental disorder of preterm infants and is associated with long-term disabilities. Early signs of cerebral palsy usually appear before three years of age. Infants with cerebral palsy compared with normal children often have slower developmental stages (28-30). Estimated prevalence of cerebral palsy in school age children is 3-4/1000 in the United States (31). Another report also showed the incidence of cerebral palsy is 2 per 1000 births in the United States (32). Although the prevalence of cerebral palsy due to infections during pregnancy and kernicterus has dropped dramatically, but the incidence preterm birth and LBW has increased dramatically which leading to emergence of a new wave of cerebral palsy especially in the developed countries. The reason is probably due to brain damage in preterm infants, parenchymal and intraventricular hemorrhage, and white matter injuries (33, 34) these factors do not apply to the full-terms (35).

Cerebral palsy, based on the type and nature of the motor disability and according to the neurological signs and symptoms, is classified into different types. The classification is also based on pyramidal or extrapyramidal pathways involved and lesion localization (quadriplegia, diplegia and hemiplegia) (36). Cerebral palsy is divided to different types such as spastics, athetoid, ataxic, or mixed paralysis (37). Spastics is the most common type of cerebral palsy in preterm infants or those with LBW;(37, 38) it includes almost two thirds of the infants born before 37 weeks of pregnancy, while for more than a century it was named as preterm disease (37). Results from different studies showed that diplegic cerebral palsy is a clinical manifestation of periventricular leukomalacia, which is from large portion damages of white matter caused by hypoxic-ischemic lesions. In other words, white matter ischemic lesions in the ventrices cause cerebral palsy especially spastic diplegia (39). Some studies have stated that premature rupture of membrane and chorioamnionitis are considered as the major risk factors for spastic cerebral palsy (37). Other studies mentioned intrauterine growth retardation as a cause for spastic diplegia. The etiology of spastic cerebral palsy is multi-factorial and it is necessary that all infants with LBW in the first two years of life be evaluated and screened regarding the neuro-motor development (19, 40). Spastic quadriplegic cerebral palsy is seen in infants discharged from the intensive care unit with a history of asphyxia, severe intraventricular hemorrhage, bilateral ventriculomegaly, neonatal seizures, and central nervous system infection (such as sepsis and meningitis) (40). In diplegia, mild to moderate cognitive impairment is observed, but it is severe in quadriplegic cerebral palsy (CP) (41). Various studies have shown that low birth weight, neonatal encephalopathy, and high risk pregnancies are considered as the most important independent risk factors for CP (42). CP develops over time; so, the symptoms may be transient and may not be the definitive consequence of cerebral palsy. On the other hand, there are infants who are discharged from the hospital with no symptoms and have had almost a normal neurological examination, especially in different kinds of diplegic or hemiplegic patients, but at the end of their first year, the peremanent signs would manifest themselves (22). In a survey conducted in Karaj, Iran, it was found that neonatal and infantile seizure, premature birth, history of abortion, and bronchopulmonary dysplasia major risk factors for developmental delays (38).

#### 3.1.2. Intellectual Disability

Intellectual disability or mental retardation (MR) is often accompanied by one or more disability states especially cerebral palsy (43). Collier SA showed that in 80.3% of the cases mental retardation is associated with cerebral palsy. In 28.2% of the patients with spastic diplegia normal intelligence is present (45). Mental retardation was observed in 4% to 5% of the children with LBW who were followed up until their school ages. Mental retardation has also been reported in severe bronchopulmonary dysplasia especially in cases that had severe and prolonged mechanical ventilation with hyperoxia (22).

Collier studied the risk factors associated with LBW and their impact on CP and mental retardation and emphasized that unwanted pregnancy and cigarette smoking during pregnancy increases the incidence of LBW; and prevention of these risk factors can prevent 32 cases of cerebral palsy and 159 cases of mental retardation per year (45).

#### 3.1.3. Hearing Loss

Studies showed that preterm infants had 20 times higher risk of hearing loss than full-terms with normal weight (46, 47). The infants' admission to the intensive care unit increases the risk of both types of sensorineural and conductive hearing loss. Therefore, it is recommended that all neonates have hearing screening before the discharge (48). LBW infants are at risk for partial or total hearing loss and approximately 2% to 3% of them suffer from this problem. Generally, all neonates, especially infants weighing less than 2000 grams are indicated for the hearing and visual evaluation after discharge (2). Valkama et al. mentioned that screening for the highly sensitive auditory brainstem response (ABR) is considered a better method for assessment of VLBW infants (48). According to the results of the hearing screening of a large number of newborns in 2013, 17.5% of the infants who had hearing loss were infants with LBW, and it was found that weight is a dependent factor in effecting decreased hearing (49). Being exposed to ototoxic drugs, infection, injury, hypoxia and ischemia, and increased serum bilirubin are also important factors in decreasing hearing in LBW infants (50).

Moderate and mild sensorineural hearing loss (25 dB to 59 dB) can lead to delayed speech and language development with the incidence of 6% to 8% of LBW infants. The high prevalence of chronic otitis with middle ear effusion and conductive hearing loss in preterm infants with LBW has been reported, and it might be due to the association with eustachian tube dysfunction, dolichocephalic head, muscular hypotonia, and prolonged intubation (22). In VLBW infants, serum bilirubin level should be kept lower than 14 mg/dL, in order to prevent cerebral palsy and sensor neural hearing loss. Also, evidence showed that increased pulmonary arterial pressure regardless of birth weight, is an independent risk factor for developing hearing impairment (51).

#### 3.1.4. Visual Impairment

Retinopathy of prematurity (ROP) is one of the most common causes of visual loss in infants with LBW (2). ROP remains as one of the causes of preventable childhood blindness worldwide and its incidence varies among different countries (52). Severe retinopathy of prematurity requires more treatment in Asia and South America than in most Western countries (53).

Gestational age of less than 26 weeks is the most important factor associated with visual impairment. It seems that hypoxia, arterial carbon dioxide pressure changes, arterial pH, oxygen consumption with high dosage, and being exposed to light are influential factors in visual defects in infants with LBW (22). Researchers studying the developmental outcomes of VLBW infants reported the prevalence of visual impairment as 5.21% and blindness in 0.5% of infants which was in one or both eyes (54). The importance of early detection of vision problems is no secret to anyone. Monitoring the eye muscle balance, vision, and visual power should be part of the premature infants follow-up programs especially up to the pre-school years. Therefore, regular follow-up and care before and after discharge in preterm and LBW infants are necessary for early detection and intervention.

#### 3.1.5. Hydrocephalus

Intraventricular hemorrhage is the major problem of premature infants in neonatal intensive care units around the world (55). According to various studies; about 15% to 20% of premature infants with birth weights lower than 1500 grams suffer from intraventricular hemorrhage and need permanent cerebrospinal fluid shunt. This figure in very premature infants with 550-750 grams of weight is approximately 45% (55-57). Prevention of intraventricular hemorrhage is critical because approximately 15% of infants after bleeding have developed hydrocephalus and 10% need shunt (57). According to recent studies, children with advanced hydrocephalus after bleeding and ventrico-peritoneal shunt in some cases had poorer neurodevelopmental outcomes (58, 59), and they would suffer from symptoms such as cerebral palsy, mental retardation, and sensory impairments (22, 56, 60, 61). Brain ultrasonography is the most reliable method for diagnosing and follow-up of intraventricular hemorrhage (IVH) in preterm infants (58, 62). With serial brain ultrasound the decrease or improvement of ventriculomegaly are shown in some cases. Clinical symptoms in infants who develop progressive hydrocephalus usually appear in weeks 2-8 or even rarely at the end of infancy (22). Since head circumference growth of more than 2 cm per week is a sign for progressive hydrocephalus (58) infant’s head circumference measurement at follow-up examinations is important; and if abnormal a Doppler ultrasound (if the increasing head circumference growth was asymmetrical with weight and height growth parameters) will be required (22).

### 3.2. Minor Impairments

Although assessment of major disabilities is easy but detecting minor impairments seems difficult (22). The rate of the presence of these defects in 6 and 24-months-old infants who were born preterm was 13.2% (63) and 11%, respectively (64). In various studies, cognitive, behavioral impairments, and neuro-behavioral developmental changes was observed with a higher prevalence in LBW and preterm infants that had survived, Frank clinical presentation appeared until the age of six years, and sometimes it was delayed until the onset of adulthood. These developmental disabilities in preterm infants include cognitive delay [low intelligence quotient (IQ)], speech and language disorders, neuro-motor disorders including problems with balance, coordination and perceptual problems, and their incidence increases with a decrease in birth weight and male gender (22, 65). In Xiong et al. study, 50% of 5-years-old children with birth age of 26-33 weeks were abnormal in more than one area of IQ, behavioral questionnaires, movement, and neurological examinations (65). Recent studies on the behavior and development of children, regardless of the type of the test used, indicate that premature infants have lower scores than full-terms (22, 63, 65). But these results can not exactly predict the prognosis for all the patients admitted to the intensive care units (22, 65).

#### 3.2.1. Perceptual and Cognitive Development

As shown in the former studies, the cognitive outcome is inversely correlated with gestational age and birth weight. Other factors that have a negative impact on cognitive outcomes are intrauterine growth retardation, perinatal infection, broncho-pulmonary dysplasia, intra ventricular hemorrhage, cystic periventricular leukomalacia, ventricular dilatation after hemorrhage, and environmental factors such as socioeconomic status, and education level of the mother. It was found that the cognitive function in preterm children were on average below term babies (67). The IQ score and cognitive and learning abilities in infants with intraventricular hemorrhage of grade four were lower. In addition, children with retinopathy of prematurity in middle childhood ages may suffer cognitive and motor impairment and have functional limitations. Bronco-pulmonary dysplasia in children with LBW and low birth age has negative impact on their IQ, mathematics and verbal ability scores, as well as cognitive and motor skills in childhood (22). Rieger study on 107 five-years-old children with birth weight lower than 501 grams and birth age of 22 weeks from three different centers in Germany showed that about 50% of the children had mild disabilities; about 41% were lagging behind in cognitive development (IQ < 70); and 80% had an IQ of 75-80 (68). Unfortunately, this defect appears in the first year of birth and gradually during the preschool years; and even with the best favorable predictor of IQ (improving IQ with better socioeconomic status), it increases. In other words, perceptual and cognitive problems in these children with average or above average social classes are more common in preterm children compared with term peers; but educating the mothers on cognitive outcomes in the second year after the child's birth should not be ignored (69). The growth and development in preterm infants via Baley scale II (BSIDII) was definitely lower than 12 months term infants which the effects of birth weight and gestational age on cognitive and behavioral outcomes (22).

#### 3.2.2. Language Development

Communication skills including auditory, visual, verbal symbolic learning systems (i.e. language), and speech production is based on academic learning and social communications (22). The prevalence of delayed or impaired language development in 3 to 5-years-old very preterm children (birth age less than 30 weeks) was between 32%-48% and in the birth age of 31-34 weeks it was 30%-35% (70). 

According to the previous studies, VLBW children and very preterm infants are weaker than term children regarding language performance. These language problems can still be seen in primary schools meaning the time that language development is more stable and becomes more like that of the adults (22). Sansavini studied 194 preterm infants and term healthy children speaking Italian language and found that the ability of preterm children in finding and combining words was significantly lower and more pathological. In this study, gender did not affect childrens' cognitive and language delay (71).

Lee in a study on 65 preterm children with 9 to 16 years of age and history of LBW and 35 full-term children with normal birth weight found that practical IQ score, verbal IQ, expressive and language skills, understanding syntax, language processing speed, verbal memory, decoding, reading, and comprehension in preterm children were significantly less advanced (72). Overall, cognitive decline in preterm infants decrease the child's ability in comprehension and receiving language, expressive language, and its parameters such as word finding (22). Riedy et al. examined 198 seven-years-old children with birth age less than 30 weeks and birth weight lower than 1250 grams and 70 full-term children with the same age. They were evaluated in terms of language skills with the use of standardized language tests. The results indicated that LBW preterm born children were weaker five areas of phonology, meaning perception, grammar, dialogue, and linguistics compared to the control group (73).

#### 3.2.3. Motor Development

Prematurity or LBW is associated with neuromotor sequels which will extend to the childhood period (69). Karimi in a case-control study on five-years-old children of Yazd, Iran, aimed to study the development of children with birth weight of 1500-2499 grams in five areas of gross and fine motor, problem solving, communication, and social and personal skills via Ages and Stages questionnaire (ASQ). He found that in this group of children, developmental delays in the areas of gross and fine motor, and problem solving were more than the children born with normal weight (74). In other studies, LBW or preterm birth was associated with poorer motor outcomes in the first and second years of life (22, 69). Significant motor impairment in very preterm and VLBW children is expected in the first year of birth until 15 years of age, although perinatal complications in children born very preterm with LBW, increases the degree of motor impairment even further in the child's life (69).

#### 3.2.4. Neurobehavioral Development

Preterm birth is the leading cause of neurobehavioral impairment and disability causing huge economic burden (75). The range of neurobehavioral impairment in preterm infants was 22%-55% compared with 7% in full-terms with the same age (69). As a portion of the preterm infants with LBW are evaluated in older ages, several potential behavioral dysfunctions are characterized in infancy and childhood (22, 68).

According to the Vieira review, nine studies have been conducted on evaluating neurodevelopment during the preschool period (3-5 years). In those studies, moderate or severe impairment of neurological function was defined as lower and upper limb motor function problems, inability to ambulate independently and visual and hearing defects. Approximately, 17%-23% of very preterm infants (birth age of less than 30 weeks) were noticed with this dysfunction in preschool stage, while it was observed in only 12% of children with birth age of 31-34 weeks. The percentage of cerebral palsy in very preterm born children at preschool was about 11%-25% and in children with birth-age of 31 to 34 weeks was 6%-15%. The majority of the risk factors of neurological dysfunction of very preterm children with LBW in preschool children were related to the neonatal period, including prolonged mechanical ventilation, grade 3 or 4 of IVH, ROP, low Apgar score, and seizure during the perinatal period. In addition to all these, low levels of maternal education was considered as one of the factors contributing to the neurological dysfunction at this age. But, the factors affecting neurological dysfunction at ages of 6-12 years, in addition to prolonged mechanical ventilation in the neonatal period, were grade 3 or 4 of IVH, periventricular leukomalacia, and mother’s age of 40 years or more at the time of delivery (70).

Early detection can lead to early interventions (75). Lund, in his study on psychological problems in three groups of adolescents with normal weight, VLBW, and small gestational age (SGA), ages of 14 and 20 years, concluded that the above problems in adolescents and young adults with LBW and preterm birth were more and often observed; although the frequency was significantly higher in SGA adolescents and young adults (76).

#### 3.2.5. School Function

With the increasing number of follow-up studies on preterm or LBW infants, it is noted that the range of disorders and learning disabilities has also a greater range (22). A meta-analysis of 14 major studies in 2009 showed that the academic achievement of children born with VLBW and observed major differences in educational attainment and poor performance in VLBW or preterm babies. In fact, this study found major differences in reading, mathematics, and spelling in VLBW (77).

Aamoudse-moens et al. studied the developmental status of academic skills in four 12-years-old children with the history of preterm birth and VLBW compared with same age full-term born children. National system of DNP (The Dutch National Pupil monitoring system) was used for evaluating the numerical reasoning and early linguistic skills in preschool stage and reading simple and complex words, comprehension, spelling, mathematics, and calculus test in primary school stage were used for evaluation. In preschool stage, very preterm children were comparable with their peer full-term in terms of basic linguistic; but in numerical reasoning skills, they were 0.7 standard deviation (SD) poorer. In the primary school, preterms were 0.3 of SD lower in reading difficult and complex words and 0.6 of SD lower in mathematics and calculus; but in terms of comprehension and spelling, they were comparable to their full-term peers. These children were more likely to renew an academic year while their academic skills would not develop. Therefore, trying to develop intervention methods can help in identifying and reducing academic weaknesses of the preterm born children (78). Five to eight years-old VLBW children were more prone to major and minor neurological dysfunction, low intelligence, poor language test performance, and low academic achievement and had more behavioral problems than their full-term peers. In addition, race, gender, and socioeconomic status did not contribute to these problems (70).

Litt also studied 181 teenagers with mean age of 14.8 years and birth weight lower than 1000 grams and discovered that these children as compared with their peer full-term children with normal birth weight had lower IQ score, lower academic achievement, and poorer functional performance and higher mathematics learning disability (79). In another study, the number of children with LBW that had not graduated from high school at the age of 19 years were four times more than those with normal birth weight (22). So, it can be concluded that surviving LBW children are at the risk of learning disabilities in school, and although they may remain in ordinary schools but they may need to repeat the school year and special training and retraining courses are required for them (68, 78, 79). Moreover, the quality of life of preterm children is lower than their peer full-term children (6-12-years-old) at school ages and at preschool years (3-5-years-old) (70).

## 4. Conclusions

The major clinical outcomes that are important for preterm infants and their families are survival and normal long term neurodevelopment. In recent years, with advances in perinatal care, the survival rate of premature infants has increased significantly, but this advancement was not associated with lower complications, and infants who survived suffered more intensely from multiple disabilities. For infants born preterm, there is now increasing evidence of sustained adverse outcomes into school age and adolescence, and the majority of them require intensive and continuous care. In line with the need to reduce infant mortality, the need to reduce the complications in premature infants should be considered by health system policymakers.

### 4.1. Strong Points of Our Study

One of the strengths of our study is the subject of study because it is a major health problem due to high mortality and chronic disorder associated with it across the world especially in developing countries such as Iran.

We found that there is a gap between the high technology and quality of acute medical care and loss or no neurodevelopmental care for high risk neonates and infants in NICUs by specialists and caregivers. No awareness of specialists and nursing of long-term neurodevelopmental outcome for high risk neonates.

### 4.2. Weak Points of Our Study

Limitation of access to journals and articles due to international sanctions against Iran.
